# Author’s Response

**DOI:** 10.1308/003588412X13373405386295

**Published:** 2012-09

**Authors:** J Joseph

**Affiliations:** Northampton General Hospital NHS Trust,UK

I read with interest your case report to which you refer in the response to our article. As you state in the report, the presence of a ‘pseudothyroid’ gland is a very rare event. Many radiologists will never come across it. However, this is a phenomenon of which clinicians need to be aware. We report the literature review that found no cases of a non-functioning thyroid with normal appearance on ultrasonography.^1^ The evidence behind this is strong and can be relied upon. We would challenge your assertion that the conclusion of the above review and therefore the statement in our article is incorrect.

Ultrasonographers who routinely scan the neck should be aware of the phenomenon you mention and account for it. This potential pitfall is not commented on anywhere in the literature, apart from your single case report, suggesting it is not a major concern. I am, however, grateful to Mr Holland for bringing to light an interesting aspect in the investigation of midline neck lumps.
